# Multilayer Substrate to Use Brittle Materials in Flexible Electronics

**DOI:** 10.1038/s41598-020-64057-6

**Published:** 2020-05-06

**Authors:** Seongmin Park, Hyuk Park, Suwon Seong, Yoonyoung Chung

**Affiliations:** Department of Electrical Engineering, Pohang University of Science and Technology, Pohang, 37673 Republic of Korea

**Keywords:** Engineering, Nanoscience and technology

## Abstract

Flexible materials with sufficient mechanical endurance under bending or folding is essential for flexible electronic devices. Conventional rigid materials such as metals and ceramics are mostly brittle so that their properties can deteriorate under a certain amount of strain. In order to utilize high-performance, but brittle conventional materials in flexible electronics, we propose a novel flexible substrate structure with a low-modulus interlayer. The low-modulus interlayer reduces the surface strain, where active electronic components are placed. The bending results with indium tin oxide (ITO) show that a critical bending radius, where the conductivity starts to deteriorate, can be reduced by more than 80% by utilizing the low-modulus layer. We demonstrate that even rigid electrodes can be used in flexible devices by manipulating the structure of flexible substrate.

## Introduction

Flexible electronic devices such as wearable sensors and flexible displays are emerging as they pioneer a new market with novel form factors^1^. For flexible electronics, materials must be capable of maintaining their properties after bending or folding. Since conventional electronic materials, which are typically metals and ceramics, are brittle, their electronic/optical properties deteriorate with a certain amount of strain. In this regard, it is essential to pave the way to manufacture flexible electronic components that are tolerable against mechanical strain for flexible devices. Numerous novel materials such as graphene^2,3^, carbon nanotube^4,5^, and conductive polymers^6^ were developed to be intrinsically unaffected by deformation. Other researchers have attempted to improve mechanical tolerance by designing a new structure with brittle materials; silver nanowires^7,8^, conductor mesh^9,10^ and multi-layer electrodes^11–14^ are typical examples of this approach. However, these approaches resulted in inferior performance, productivity and process compatibility, compared to the brittle conventional materials. Researchers also studied the use of conventional materials in flexible electronic devices by manipulating the location of neutral plane with additional layers on top^15–18^. However, this methodology has focused on embedding active components between polymeric materials with a proper thickness and Young’s modulus. In addition, their approach was to place the device on the neutral plane accurately, which is difficult to be adopted in multilayer structure. In other studies, a soft material was sandwiched between relatively stiff materials, so that the soft layer can reduce the surface strain^19^. This approach yet required flexible electronic materials and was difficult to be applied in current electronic device fabrications. For flexible optoelectronic applications, there have been much efforts to increase the flexibility of brittle indium tin oxide (ITO);^20–22^ however, these methods included complicated and high-priced processes. MICAtronics utilizes mica substrate to make flexible devices with van der Waals epitaxy^23–26^. Optoelectronic devices on mica showed good endurance under 10^4^ bending cycles at bending radius of 2 mm^23,24^. However, weak adhesion between the substrate and device layers remains challenges for multiple fabrication steps, and it is yet large-area compatible.

In this work, we introduce a low-modulus layer inside a regular flexible substrate to utilize brittle materials in flexible electronics, rather than developing novel materials. When a low-modulus layer is inserted, two additional neutral planes are formed near the materials interface^27,28^. If the neutral plane is located closer to the surface, the strain is reduced on the surface, where active components are located, compared to the device without the interlayer. By increasing the thickness ratio of the low-modulus material, the outer neutral planes move closer to the surface, so that the surface strain can be further reduced while bent. We demonstrate this novel flexible substrate concept with brittle materials such as ITO and nickel. Brittle conductors on flexible substrate with low-modulus interlayer exhibited much less resistance change while bent, compared to a regular homogeneous substrate. Through cyclic bending tests, finite element method simulations and angular-dependence measurements, the effects of multilayer flexible substrate were studied, and the brittle materials showed much improved flexibility.

## Results

**Figure 1:**
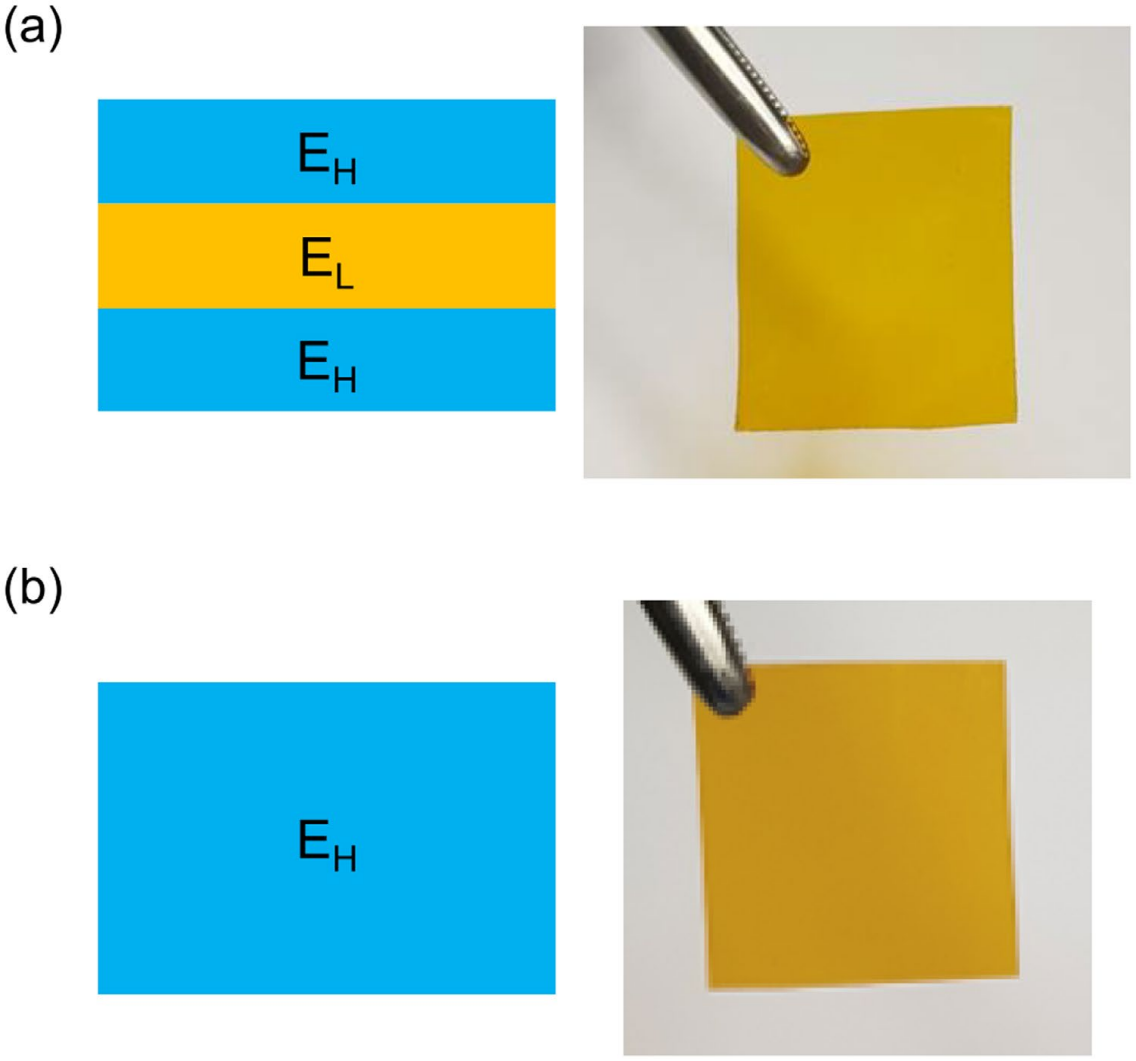
The cross-sectional structure and photograph of two types of flexible substrates: (**a**) Trilayer and (**b**) Monolayer. The Monolayer substrate is composed of high-modulus material (E_H_) only, whereas the Trilayer substrate contains a low-modulus interlayer (E_L_) in between the high-modulus layers.

We fabricated three types of flexible substrates to study the effects of low-modulus interlayer under bending condition. One was a regular homogeneous flexible substrate, denoted as ‘Monolayer.’ The others were trilayer flexible substrates, denoted as ‘Trilayer 1’ and ‘Trilayer 2’, where low-modulus interlayer (E_L_) was sandwiched in between high-modulus layers (E_H_). The total thickness of each substrate was fixed to be 130 µm. In the Trilayer 1, the thickness of each layer was E_H_:E_L_:E_H_ = 40:50:40 µm. The Trilayer 2 had thicker E_L_, and the thickness of each layer was E_H_:E_L_:E_H_ = 20:90:20 µm. The fabrication process of the multilayer flexible substrate is depicted in Supporting Information Fig. S1 in details. Polyimide (PI), widely adopted in industry, was used as a high-modulus material, and polydimethylsiloxane (PDMS) was used as a low-modulus material. The structure and photograph of the trilayer and monolayer substrates are shown in Fig. 1.

**Figure 2:**
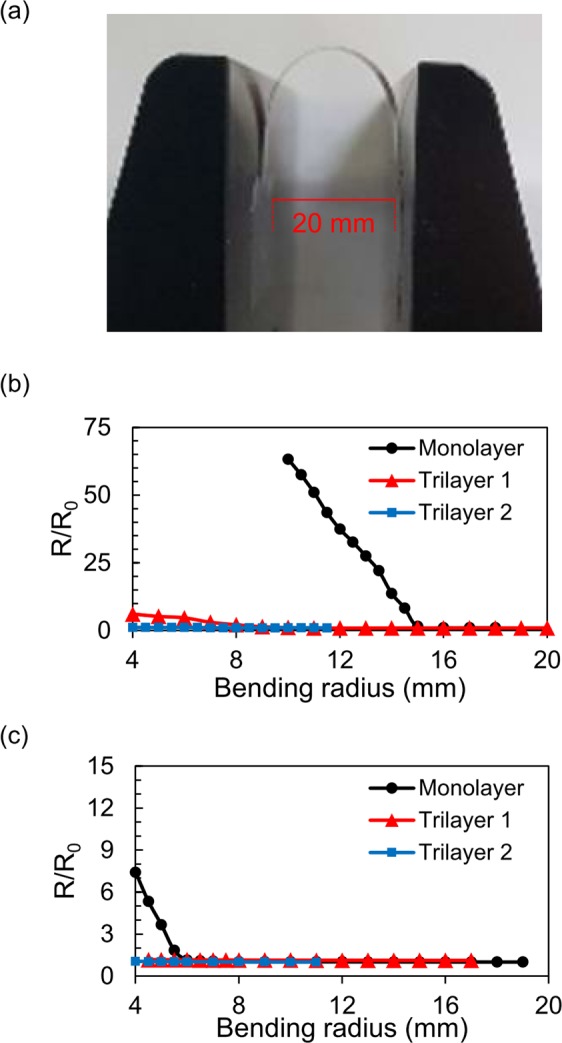
Bending test of brittle thin films on Monolayer and Trilayer substrates. The probe tips were aligned perpendicular to the bending axis. (**a**) A photograph of cyclic bending test machine. Normalized sheet resistance vs. bending radius of each flexible substrate (**b**) with ITO (100 nm) and (**c**) with Ni (100 nm) on top. (**b**,**c**) The structure of Monolayer and Trilayer substrates is shown in Fig. 1, and the total thickness of each substrate was fixed to be 130 µm. In the Trilayer 1, the thickness of each layer was E_H_:E_L_:E_H_ = 40:50:40 µm. The Trilayer 2 substrate had thicker E_L_, and the thickness of each layer was E_H_:E_L_:E_H_ = 20:90:20 µm. The results show that the low-modulus interlayer (E_L_) effectively reduces the surface strain on top so that a mechanical bending is less effective in the Trilayer samples. As the thickness of E_L_ increases, a tolerance against mechanical strain is improved.

We conducted bending tests to compare the mechanical tolerance of brittle thin-film electrodes between the three types of flexible substrates. We deposited 100-nm-thick ITO or nickel on the three substrates, as they are representative brittle conducting materials^29^. After deposition, we detached the substrate from the carrier wafer and mounted it on a bending machine. The bending test equipment used for this experiment is shown in Fig. 2a, and a schematic of the sample is shown in Supporting Information Fig. S1. After bending with a certain radius, the sheet resistance was monitored by 4-point probe measurement. As the bending radius was decreased, the resistance value was increased from the initial value (R_0_), measured without bending. As shown in Fig. 2b,c, the electrode on the Trilayer substrates maintains its resistance until much lower bending radius than the Monolayer sample. While bending towards lower radius, the ITO electrode on the Monolayer substrate started to increase the resistance at a bending radius of 15 mm. Whereas, the resistance of ITO on the Trilayer 1 was increased at a bending radius 9 mm, and the resistance remained unchanged until a radius of 4 mm on the Trilayer 2 substrate. We note that 4 mm was the well-controlled minimum bending radius with the test machine. In case of the nickel electrode, as shown in Fig. 2c, the Monolayer sample exhibits a resistance increase from a bending radius of 6 mm, while the Trilayer 1 and 2 samples maintains the same performance until the minimum bending radius of 4 mm. These results demonstrate that the low-modulus interlayer can effectively reduce the surface strain while maintaining the sample thickness. Moreover, as the relative thickness of the low-modulus interlayer increases, the strain on the top surface is reduced when bent, and the upper electrode can be further tolerable against bending.

**Figure 3:**
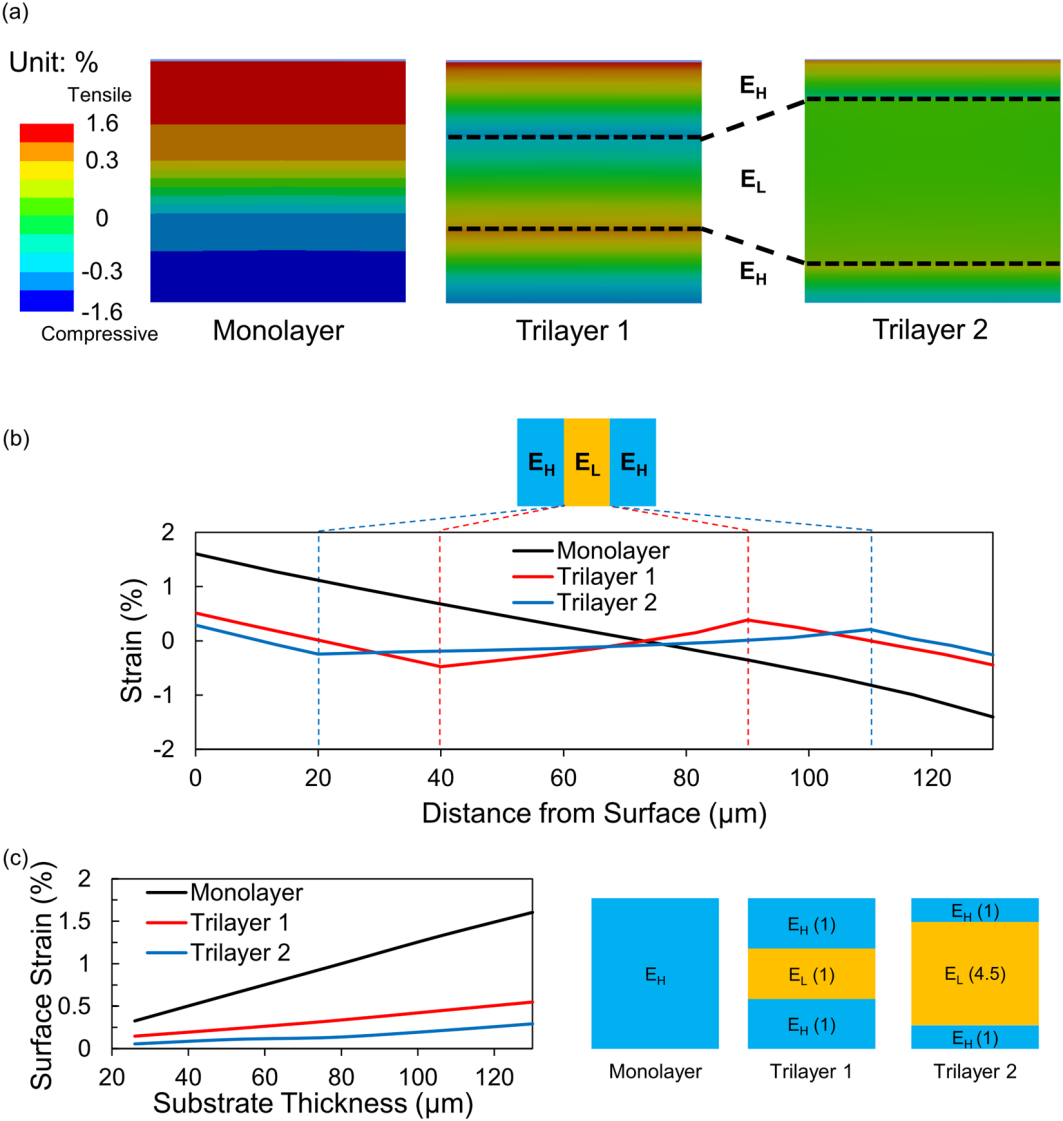
Finite element method simulation results of bending strain inside the flexible substrates when they are bent with a bending radius of 4 mm. (**a**) Cross-sectional strain distribution in the three flexible substrates. (**b**) Strain data as a function of the distance from the substrate surface. In the Monolayer substrate, the strain is distributed linearly with one neutral plane near the middle of the film. In the Trilayer substrates, however, two additional neutral planes are formed, and the strain curves are deviated from the linear Monolayer data. As additional neutral planes locate near the interface between E_H_ and E_L_, the surface strain is reduced in the Trilayer samples. (**c**) Surface strain value as a function of substrate thickness at a bending radius of 4 mm. The diagram on the right shows the thickness ratio of each layer. (Drawn by Microsoft Powerpoint 2016 https://products.office.com/en-us/home).

To analyse the mechanism for the enhanced flexibility of brittle conductors on the Trilayer substrate, we conducted finite element method simulations. The three types of flexible substrates were bent with a bending radius of 4 mm, and the strain value was calculated inside the substrate. In Fig. 3, the simulation results show that the strain distribution in the Monolayer substrate is almost linear from the bottom to the top surface. However, in the case of the Trilayer substrates, the strain graph exhibits a local maximum or minimum, where its first derivative is zero, at the interface between the high and low modulus layers, and additional neutral planes are formed in the high-modulus layers. In the Trilayer 1, the surface strain is reduced by 68%, compared to the Monolayer substrate; in the Trilayer 2, which has higher ratio of the low-modulus layer, the surface strain is more reduced by 82% than the Monolayer. We confirmed that the low-modulus layer generates additional neutral planes near the surface, and they lead to the reduced surface strain when bent. In addition, as the portion of the low-modulus layer increases, the neutral planes are formed closer to the surface, which in turn results in further reduced surface strain.

The film thickness is a major factor affecting the strain value, along with the modulus of flexible substrate. We conducted FEA simulations to check the correlation between the surface strain and the substrate thickness (see Fig. 3c). In this simulation, the samples were bent with a radius of 4 mm, the thickness ratio of E_H_ and E_L_ was fixed, and the total thickness was varied. The results confirm that the thickness of flexible substrate can be increased while maintaining the same amount of surface strain in the Trilayer.

**Figure 4:**
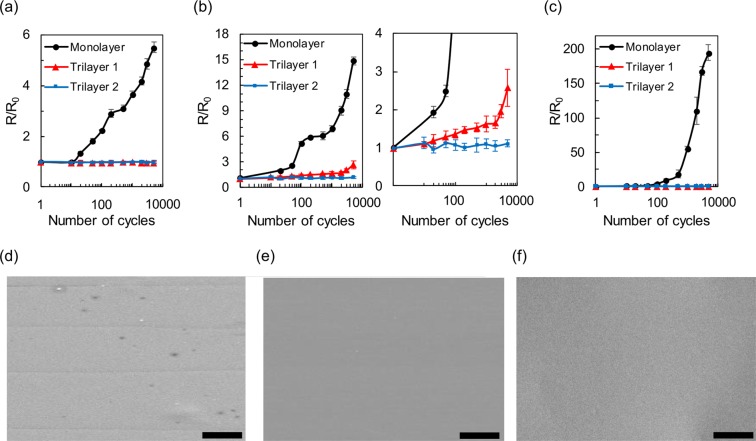
Cyclic bending test results of brittle thin-film conductors. Normalized sheet resistance of ITO (100 nm) on the Trilayer and Monolayer substrates as a function of consecutive bending cycles with bending radius of (**a**) 11 mm (**b**) 10 mm. The right figure in (**b**) is an enlarged view of the left. (**c**) Normalized sheet resistance of nickel (100 nm) on the Trilayer and Monolayer substrates with a bending radius of 4 mm. The probe tips were aligned perpendicular to the bending axis. The results show that the durability against bending stress can be greatly increased by using the low-modulus layer inside the flexible substrate. Scanning electron microscope (SEM) images of nickel thin film after 100 bending cycles with a bending radius of 4 mm (**d**) on the Monolayer substrate; (**e**) on the Trilayer 1 and (**f**) on the Trilayer 2. The bending axis is in horizontal direction, and the scale bar indicates 10 μm. The effects of Trilayer samples are corroborated with the four linear cracks, parallel to the bending axis, in (**d**). The nickel electrode on the Monolayer substrate was cracked after 100 cycles, but no crack was observed the Trilayer samples after the identical bending condition.

For practical flexible electronic applications, flexible electrodes must be capable of maintaining their properties even after a large number of bendings and releasings. To cheque the endurance of brittle electrodes on the Monolayer and Trilayer substrates, we conducted cyclic bending tests of ITO and nickel thin films (100 nm) at various bending radii. In Fig. 4a with a bending radius of 11 mm, an ITO thin film on the Monolayer shows a significant resistance increase, while the Trilayer samples exhibit almost no degradation until 5,000 bendings and releasings. With a bending radius of 10 mm in Fig. 4b, the ITO on the Trilayer 2 maintains its resistance at 5,000 cycles; however, the Monolayer and Trilayer 1 samples shows evident degradation. For the nickel thin film, the bending radius was set to be 4 mm, which was the minimum available value with the cyclic bending equipment. As shown in Fig. 4c, the nickel electrode on the Trilayer substrates exhibits no resistance change until 5,000 cycles, whereas the performance of Monolayer sample degrades rapidly at only 10 cycles. The cyclic bending tests confirm that the Trilayer substrate effectively enhance the endurance of brittle conductors under consecutive bending condition; moreover, the bending endurance can be improved as the ratio of low-modulus layer is increased in the Trilayer substrate.

The reduced strain on the Trilayer substrate was corroborated by scanning electron microscope (SEM) images in Fig. 4d–f. The resistance increase of the brittle thin film under bending condition is known to be caused by the formation of cracks parallel to the bending axis^30^. Before any bending, no crack was observed in the nickel thin film (100 nm) on the Monolayer and Trilayer substrates (see Supporting Information Fig. S2). After 100 bending cycles with 4 mm bending radius, linear cracks were found on the Monolayer sample as shown in Fig. 4d. However, such crack was not observed in the Trilayer samples under the same bending condition (see Fig. 4e,f), which is a direct evidence that the low-modulus interlayer reduces the surface strain. This result is consistent with the cyclic bending test in Fig. 4c, where the Trilayer samples show no resistance change even after 100 bending cycles.

**Figure 5:**
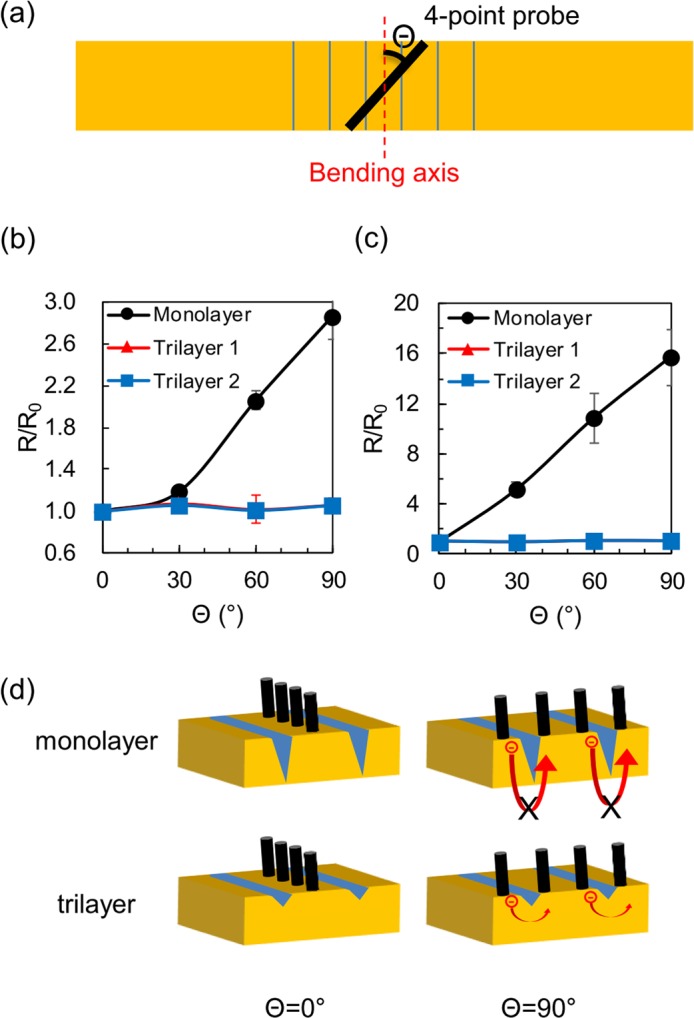
Sheet resistance measurements on ITO and nickel thin films (100 nm) with different angles. (**a**) Schematic top view of the angle Θ between the bending axis and the direction of 4-point probe tips. (**b**) Normalized sheet resistance of ITO vs. Θ after 5,000 bending cycles with bending radius of 10 mm, where R_0_ is the sheet resistance at Θ = 0°. (**c**) Normalized sheet resistance of nickel vs. Θ after 5,000 bending cycles with bending radius of 4 mm. **(d**) Schematic image describing the different angular dependency between the Monolayer and Trilayer substrates. As the Θ increases, the movement of electrons becomes more disturbed by the cracks, which can increase the sheet resistance. On the Trilayer samples, however, shallower cracks, due to the reduced surface strain, have negligible effects on the conduction and thus result in much less angular dependency, compared to the Monolayer sample. (Drawn by Microsoft Powerpoint 2016 https://products.office.com/en-us/home).

We measured the angular dependence of sheet resistance on ITO and nickel thin films (100 nm) after consecutive bendings. The brittle thin films were made onto the three types of flexible substrates, bent/released 5,000 times and measured by 4-point probe. The angle between the 4-point probe tips and the bending axis (Θ) is shown in Fig. 5a. In case of the ITO samples (Fig. 5b), the bending radius was fixed to be 10 mm, and the sheet resistance of the Monolayer sample was increased by more than 2.5 times while the Θ changed from 0° to 90°. Whereas, the resistance of the Trilayer samples exhibits no angular dependence. The nickel samples were bent with a bending radius of 4 mm, and they showed similar results as ITO. As shown in Fig. 5c, the Monolayer sample had a significant resistance increase by more than 10 times when the Θ changed from 0° to 90°. The Trilayer samples, however, do not show any angular dependence. We attribute these results to the surface strain and resultant cracks on the brittle thin films. When Θ = 0°, the sheet resistance is the smallest as the effect of the linear crack is insignificant with 4-probe measurements (see Fig. 5d). As the Θ increases, the current flowing between 4-probe tips is disturbed by the cracks; this interference is the largest at Θ = 90°. Because the Monolayer samples contain deeper cracks, they show large angular dependence, compared to the Trilayer samples with shallower or negligible cracks. These results correspond to the cyclic test data in Fig. 4.

## Discussion

In summary, we demonstrate that the flexibility of thin-film device can be much improved by engineering the flexible substrate. The low-modulus layer inside flexible substrate effectively reduces the surface strain when bent, thereby enabling the use of even brittle materials in flexible electronics. A critical bending radius of ITO thin film (100 nm) on flexible substrate (130 µm) was reduced by more than 70% with this approach. The effects of multilayer flexible substrate were analysed with cyclic bending test, finite element method simulations, SEM images and angular measurement. Instead of developing novel flexible materials, our study provides a high degree of freedom in materials selection for flexible electronics.

## Methods

### Fabrication and measurement

The overall sample fabrication is depicted in Supporting Information Fig. S1. We used polyimide (IPI-N, IPITECH) as high-modulus and PDMS (Sylgard 184, Dowcorning) as low-modulus materials. An ITO thin film was deposited by RF sputter system and annealed at 200 °C for 4 hours under N_2_ condition. A nickel thin film was deposited by E-beam evaporator. The sheet resistance was measured by using semiconductor analyzer (B1500A, Keysight) and 4-point probe tips with 10 μm distance.

### Simulation

The finite element method simulations were performed using the ANSYS Mechanical program. The Young’s modulus (E) and Poisson ratio (ν) were E_PI_ = 1.94 GPa and ν_PI_ = 0.34 for PI^31^; E_PDMS_ = 3.27 MPa and ν_PDMS_ = 0.48 for PDMS^32^. The modulus of PI and PDMS was measured by the stress-strain curve in Supporting Information Fig. S3.

## Supplementary information

Supplementary information.
