# Supplementary material for: Multilayer Substrate to Use Brittle Materials in Flexible Electronics
Source: Sci Rep. 2020 May 6;10:7660. doi: 10.1038/s41598-020-64057-6 (PMC7203148; doi:10.1038/s41598-020-64057-6)
Supplement: Supplementary file 1 — Supplementary information. [file 41598_2020_64057_MOESM1_ESM.docx]

Multilayer Substrate to Use Brittle Materials in Flexible Electronics-Supplementary Information

Seongmin Park^1^, Hyuk Park, Suwon Seong^1^, and Yoonyoung Chung^1^*

^1^ Department of Electrical Engineering, Pohang University of Science and Technology

Pohang 37673, Republic of Korea

*E-mail: ychung@postech.ac.kr


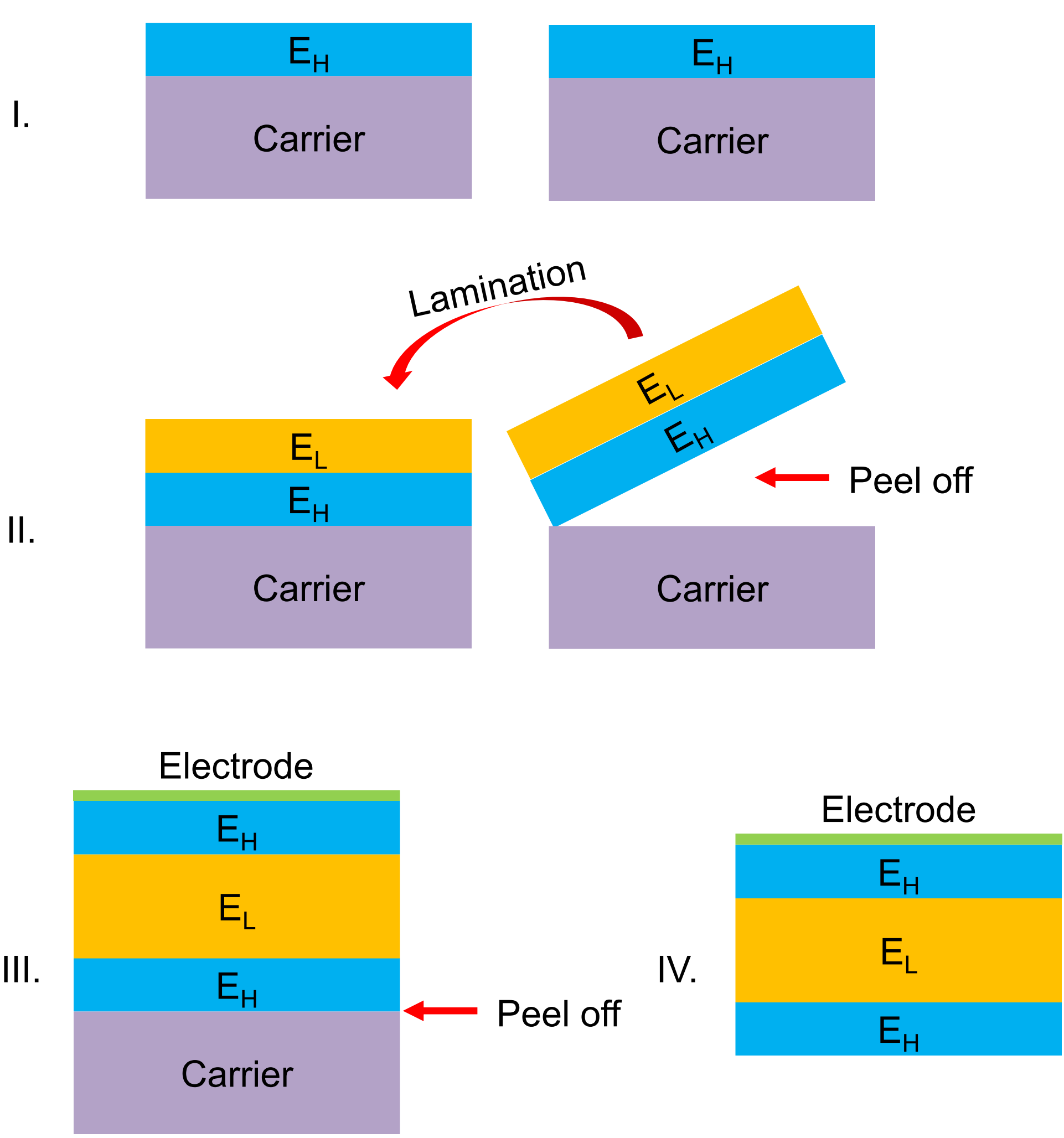


Figure S1 | The fabrication process of multilayer substrate. I) Spin coating of PDMS on PI and partial curing of PDMS at 60 ℃ for 35 min. II) Peeling of one PI/PDMS sample and lamination onto the other PI/PDMS. III) Deposition of thin-film electrode on top. IV) Final sample structure after peeling off from carrier substrate. (Drawn by Microsoft Powerpoint 2016 https://products.office.com/en-us/home)


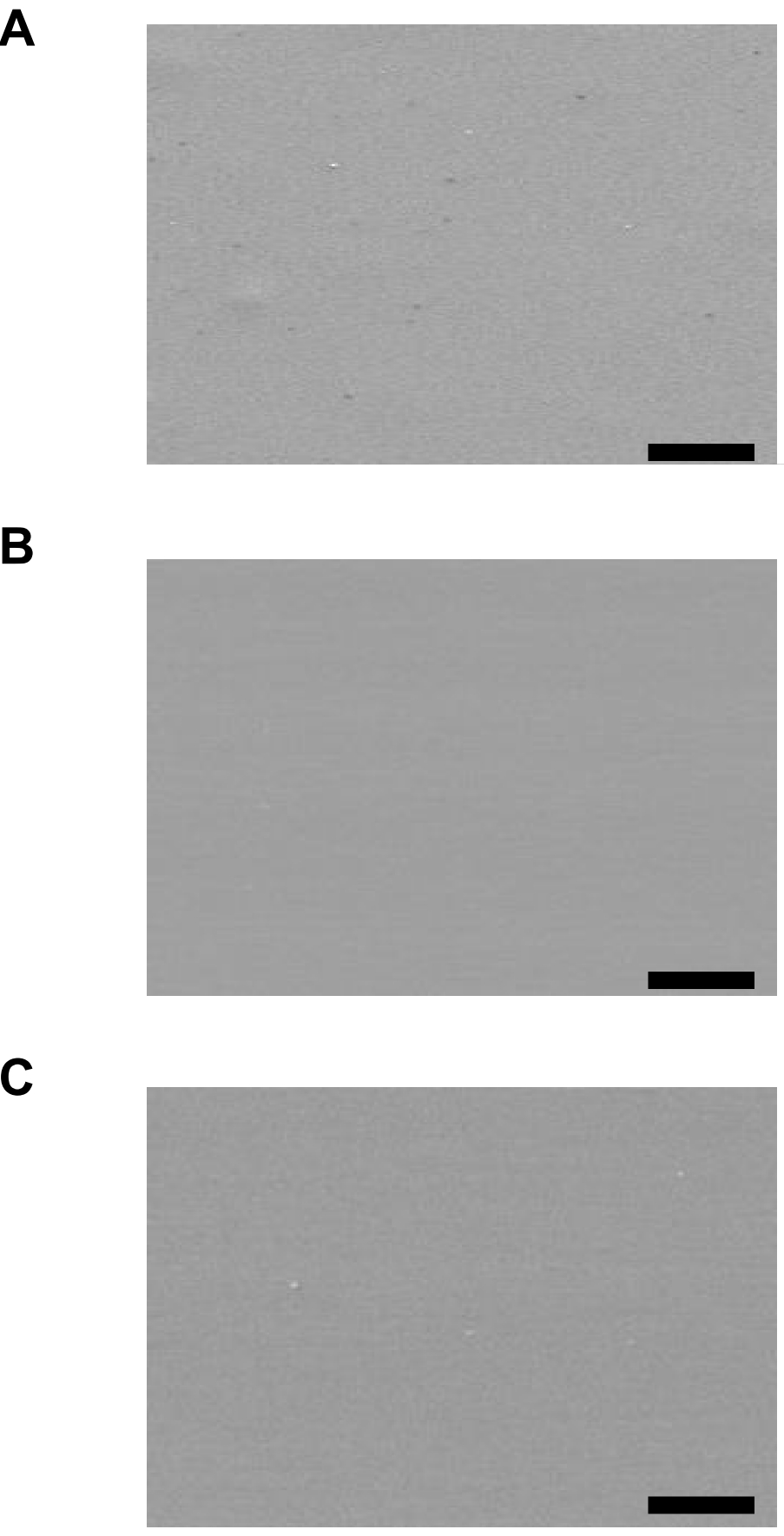


**Figure S2 | Scanning electron microscope (SEM) images of nickel thin film (100 nm) on (a) Monolayer, (b) Trilayer 1 and (c) Trilayer 2 substrates before cyclic bending test.** The scale bar indicates 10 μm. No crack is observed before bending.


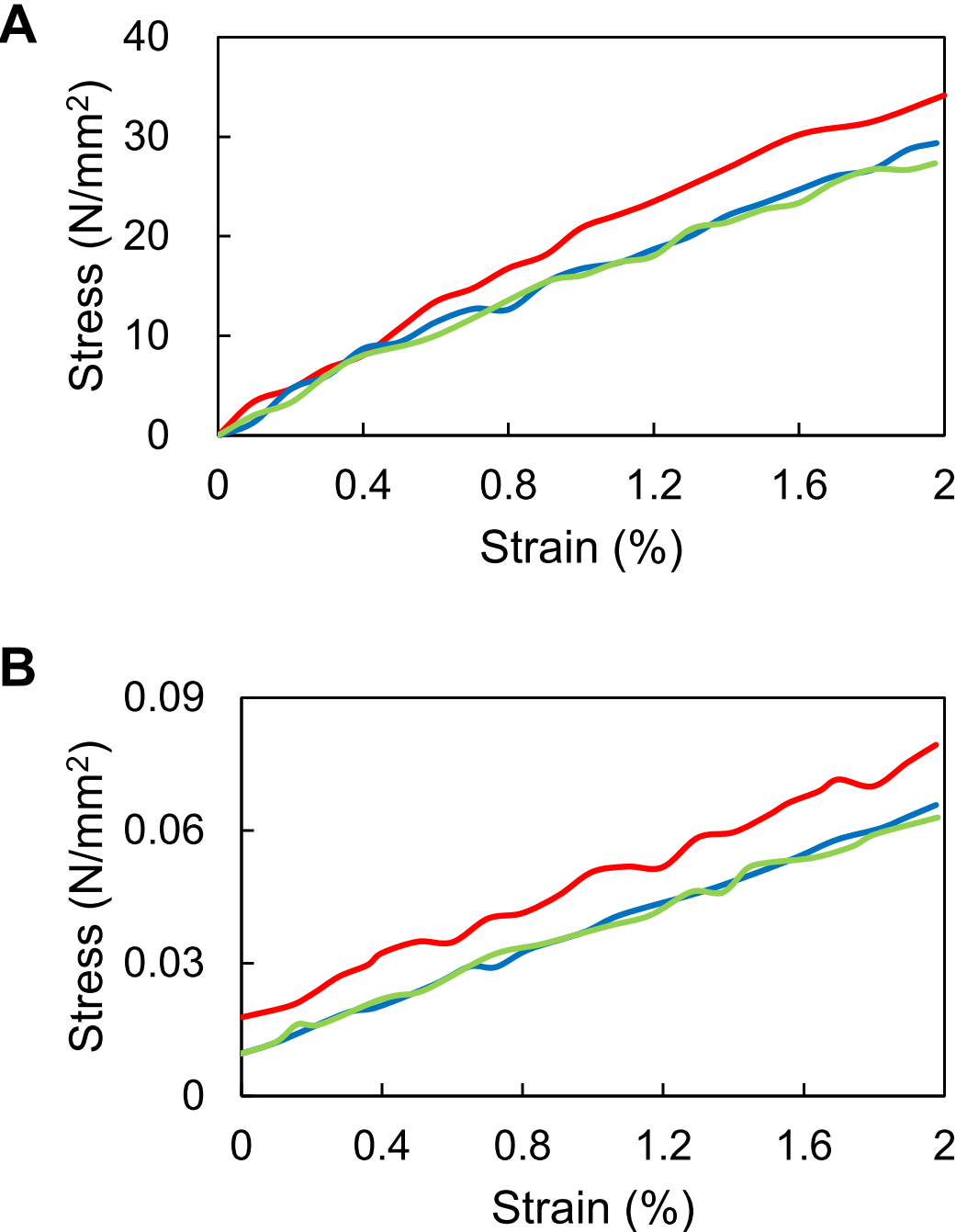


**Figure S3 | The stress-strain curve of (a) PI and (b) PDMS.** The measurements were done with a universal testing machine, and the load cell was 200 N with a moving speed of 5 mm/min. From these measurement results, the Young’s modulus of PI and PDMS was estimated to be 1.94 GPa and 3.27 MPa, respectively.
